# Ligand Binding Induces Agonistic-Like Conformational Adaptations in Helix 12 of Progesterone Receptor Ligand Binding Domain

**DOI:** 10.3389/fchem.2019.00315

**Published:** 2019-05-07

**Authors:** Liangzhen Zheng, Kelin Xia, Yuguang Mu

**Affiliations:** ^1^School of Biological Sciences, Nanyang Technological University, Singapore, Singapore; ^2^School of Physical and Mathematical Sciences, Nanyang Technological University, Singapore, Singapore

**Keywords:** progesterone receptor, ligand binding domain, conformational changes, molecular dynamics, helix 12

## Abstract

Progesterone receptor (PR) is a member of the nuclear receptor (NR) superfamily and plays a vital role in the female reproductive system. The malfunction of it would lead to several types of cancers. The understanding of conformational changes in its ligand binding domain (LBD) is valuable for both biological function studies and therapeutically intervenes. A key unsolved question is how the binding of a ligand (agonist, antagonist, or a selective modulator) induces conformational changes of PR LBD, especially its helix 12. We applied molecular dynamics (MD) simulations to explore the conformational adaptations of PR LBD with or without a ligand or the co-repressor peptides binding. From the simulations, both the agonist progesterone (P4) and the selective PR modulator (SPRM) asoprisnil induces agonistic-like helix 12 conformations (the “closed” states) in PR LBD and the complex of LBD-SPRM is less stable, comparing to the agonist-liganded PR LBD. The results, therefore, explain the partial agonism of the SPRM, which could induce weak agonistic effects in PR. We also found that co-repressor peptides could be stably associated with the LBD and stabilize the LBD in a “semi-open” state for helix 12. These findings would enhance our understanding of PR structural and functional relationships and would also be useful for future structure and knowledge-based drug discovery.

## Introduction

As a member of the NR superfamily, PR is involved in versatile important biological functions, especially female development, reproduction and maintenance (Mangelsdorf et al., 1995; Huang et al., 2010). There are majorly two sub-types of PR, PRA and PRB, transcribed and translated from the same gene but with different promoters (Kastner et al., 1990; Vegeto et al., 1993). These two types of PR differ in the length of the amino acid sequences, where PRB has full-length sequence and PRA lacks a 164 residue N-terminal part (Spitz, 2005; Hill et al., 2012). Binding of P4 (or other progestins) in the PR ligand binding pocket (LBP) would induce conformational changes of PR LBD and homo-dimerization (two same PR isoforms) or PRA-PRB hetero-dimerization in PR positive cell cytosol (Spitz, 2005).

The dimer then enters the nucleus and binds to specific DNA sequences, the PR response elements (PREs), and recruits co-activator proteins, such as steroid receptor co-activator-1 (SRC-1) and CREB-binding protein (CBP) (Williams and Sigler, 1998; Liu et al., 2002; Spitz, 2003), thus facilitates the subsequent gene expressions (Spitz, 2005). Whereas, binding of an antagonist or SPRM would also induce PR dimerization and the relocation from cell cytosol to the nucleus, but would induce co-repressor binding with the activation function 2 (AF2), thus prohibit the normal gene expression functions (Spitz, 2005; Madauss et al., 2007).

The overall structure of PR LBD resembles the general NR LBD conformations (Bain et al., 2007). The early X-ray crystallography studies indicate that with agonists binding, PR LBD adopts the agonistic conformation (the “closed” state), where it forms the classic sandwich folding scheme with helix 12 tightly patched on the LBP (Williams and Sigler, 1998). Hydrogen bonds between M909 in the N-terminal part of helix 12 and E723 (in helix 3) hook helix 12 in the “closed” state, meanwhile more hydrogen bonds formed between the loop linking helix 11 and helix 12 (named H11–H12 loop hereafter) and helix 3 further stabilize the “closed” conformation (Williams and Sigler, 1998; Petit-Topin et al., 2009). SPRM binding in the LBD would induce different conformational changes (Madauss et al., 2007; Raaijmakers et al., 2009; Lusher et al., 2011, 2012; Petit-Topin et al., 2014). The ligand soaking experiments of PR LBD with SPRM asoprisnil (Lusher et al., 2012) and antagonist RU486 (Raaijmakers et al., 2009) binding witness the unstable agonistic conformations of PR LBD. In those PR LBD crystal structures, helix 12 covers on the LBP and adopts the AF2 activation state, though helix 12 has rather large b-factors. In other scenarios, SPRM-bound PR LBD would adopt the antagonistic conformation (the “open” state) and recruits co-repressors, such as nuclear corepressor (NCoR) and silencing mediator retinoid and thyroid hormone (SMRT), binding in the hydrophobic cleft formed by helix 3 and helix 5 (Chen and Evans, 1995; Leonhardt and Edwards, 2002; Liu et al., 2002; Spitz, 2003; Madauss et al., 2007; Petit-Topin et al., 2014).

The deposited crystal structures of PR LBD in RCSB Protein Data Bank (PDB) all adopt a typical 11 helices folding scheme as other LBDs in NR family (Bain et al., 2007). All the structures are *holo*-form, either with a ligand or together with a co-peptide (a segment of SMRT co-repressor peptides), no *apo*-form PR LBD conformations have been determined using X-ray or NMR methods. Our previous theoretical study (Zheng et al., 2016) has proved that, for *apo*-form PR LBD, there exists multiple intermediate states, which include the agonistic conformations (the “closed” states) with helix 12 hooked by helix 3 (Williams and Sigler, 1998; Petit-Topin et al., 2009), forming a solvent inaccessible isolated LBP.

Though no experimental models have been solved for the structures of the *apo*-form PR LBD, computational simulation study and time-resolved fluorescent anisotropy decay method have proved that the helix 11, H11–H12 loop and helix 12 are highly flexible (Batista and Martinez, 2013; Zheng et al., 2016). Helix 12 was observed to form a totally extend conformation in the *apo*-form of αER and αRXR LBDs (Tanenbaum et al., 1998; Batista and Martinez, 2013), it is not clear whether the longer helix 12 in PR LBD takes such an ordered configuration.

The biological effects of some of PR LBD binding ligands have been studied thoroughly. There are majorly three types of PR LBD ligands: agonists, antagonists and SPRMs. P4 and other progestins, though have different pharmacological profiles and effects (Sitruk-Ware, 2004; Sitruk-Ware et al., 2004), they generally activate the functional gene expressions, repress estrogen induced endometrial proliferation, and inhibit the endometrial mitogenic effects of estrogens (Vegeto et al., 1993; Kraus et al., 1995; Schindler et al., 2003). Another type of PR LBD ligands, the SPRMs, such as asoprisnil (DeManno et al., 2003), would in some case bring the partial agonism as observed in clinical trials and animal tests (Elger et al., 2000; Spitz, 2003; Chwalisz et al., 2005a,b). The third group of ligands the antagonists of PR LBD, such as RU486 and APU (Spitz, 2003; Petit-Topin et al., 2014). The antagonists pose inhibitory effect against biological active PR LBDs and would be used for pregnancy termination and the treatment of certain PR mediated conditions.

So far there are lots of unknowns for the liganded structural features of PR LBD. For example, how are agonistic and antagonistic conformations formed or stabilized by different ligands? We hypothesize that agonists or SPRMs binding would induce conformational adaptation of PR LBD through re-positioning helix 12 in the “closed” agonistic conformation, and the additional co-repressor peptides would stabilize the “open” antagonistic conformation together with SPRMs. MD simulations were adopted to explore the atomic level mechanisms of PR LBD conformational dynamics. We found that ligand binding would stabilize PR LBD in “closed” state, and both agonist and SPRM binding would induce the “open-to-closed” transition of the LBD involving the repositioning of helix 12 in an agonistic-like orientation. The detailed ligand-induced conformational transition of PR LBD could be divided into three steps: hydrophobic cluster forming, helix 12 patching and H11–H12 loop re-accommodation. And the driving forces of the ligand-induced conformational adaptation are also discussed. The atomic-level understanding of the agonist P4 and SPRM asoprisnil induced conformational adaptations explains the partial agonism of SPRM, because it binds PR LBD inducing unstable agonistic “closed” conformations of helix 12. These findings broaden our knowledge of the structure-function relationship of LBD-ligand complex system, and also feeds future knowledge-based drug design against PR LBD.

## Materials and Methods

### Simulation Protocols

Molecular dynamics simulations are useful tools to explore molecular behavior and mechanism in atomic level. In this study, we harness the power of graphic processing unit (GPU) cards to perform micro-second range conventional MD simulations to understand the determinants of the PR LBD conformation changes. Several systems were built to study the protein conformation/dynamics in the presence of ligands and/or co-peptides (a segment of SMRT co-repressor peptides), as listed in the Table 1.

**Table 1:** Summary of the simulation systems in this study.

**Simulation systems**	**LBD initial**	**Ligand**	**Co-peptides**	**# of Repeats**	**Simulation Time (ns)**
S1	1A28, chain A	P4	None	1	2,000
S2	1A28, chain A	None	None	1	2,000
S3	2OVH, chain A	Asoprisnil	None	3	2,000
S4	2OVH, chain A	None	None	1	2,000
S5	2OVH, chain A	None	2OVH, chain B	1	2,000
S6	2OVH, chain A	P4	None	1	1,000
S7	2OVH, chain A	Asoprisnil	2OVH, chain B	1	2,000

Initial structures of the LBD, ligands and co-peptides were downloaded from RCSB protein data bank (PDB) (see Table 1). Original water molecules in the PDB structures were removed, missing atoms and residues of LBD were modeled in SWISS-MODEL online server (https://swissmodel.expasy.org/; Schwede et al., 2003). For simulation system S6, the initial LBD-ligand complexes were modeled by removing the original ligand from LBP and docking P4 into LBD using GOLD docking package (Verdonk et al., 2003). The docking calculations of P4 based on the 2OVH chain A, without co-peptide binding, using the default parameters for GOLD, and the best docking pose together with the LBD receptor was selected for initial structure for system S6.

The amber99sb-ildn force field (Lindorff-Larsen et al., 2010) was used for LDB and co-peptides. As for ligands, AM1-bcc charges together with amber gaff force field were used (Wang et al., 2004). The Gromacs format ligand topology files could be freely accessible in Figshare (https://figshare.com/articles/Small_molecular_GAFF_force_field_files/7982333). The LBD, or the LBD in complex with ligand or co-peptides were solvated in abundant TIP3P (Mark and Nilsson, 2001) water molecules as well as 0.15 M NaCl salt condition. All simulations were completed with Gromacs 5.1.2 package (Abraham et al., 2015) together with GPU acceleration on National Supercomputing Center (NSCC), Singapore (https://www.nscc.sg/).

Each simulation system was firstly energy minimized using the steepest descent algorithm, and then equilibrated with protein (as well as ligands if exists) heavy atom position constrained using a 1,000 kJ/mol·nm^2^ force constant under *NPT* ensemble at 300 K and 1 bar for 10 ns. Production runs were performed under *NVT* ensemble at 300 K. The time step for all simulations was 2 fs, while the coordinate data were stored every 2 ps. Thermostats and pressure coupling were achieved using velocity rescaling (Bussi et al., 2007) and Berendsen pressure coupling (Berendsen et al., 1984) method, respectively. Bonds between heavy (non-hydrogen) atoms were restricted with LINCS algorithm (Hess et al., 1997), while bonds between hydrogen atoms and heavy atoms were fixed according to SHAKE algorithm (Ryckaert et al., 1977). Particle mesh Edwald (PME) scheme (Darden et al., 1993) algorithm was adopted for the long-range electrostatic potential calculation. A 1.2 nm distance cutoff for both long-range electrostatic and van der Waals interactions was used.

### Analysis Methods

*ΔRMSD*, used in one of our previous studies (Zheng et al., 2016), is defined by the difference between two root mean square deviation (RMSD) values, RMSD1 and RMSD2, which are the instance RMSD calculated using crystal “open” state (PDB ID 2OVH, chain A) and “closed” state (PDB ID 1A28, chain A) LBD conformations as references.

The principle component analysis (PCA) were performed using python scikit-learn package and numpy package (Walt et al., 2011). The alpha carbon (αC) atom distance matrices were used as datasets for PCA analysis. For one simulation trajectory, the conformations of every 100 ps were collected, and the distances between all αC atoms were calculated. The dataset thus was fed into the scikit-learn decomposition module, and the transformed dataset was plotted. Based on the PCA analysis, the eigenvectors per αC atom were determined and adopted for the essential dynamics of each residue and visualized using VMD 1.9 (Humphrey et al., 1996).

Clustering analysis were performed using Gromacs g_cluster tool with gromos algorithm and a 0.2 nm RMSD cutoff. Quasi-Harmonic conformational entropy (Levy et al., 1984) calculations of PR LBD were performed using Gromacs g_covar and g_aneig tools. The method assumes that there exists a harmonic multi-variant probability distribution of all 3*N* atoms in the calculation system, where *N* represents the number of atoms in a molecule. Similar method has been applied for the flexibility proximation of different macromolecule systems (Williams and Maher, 2000; Okonogi et al., 2002; Podesta et al., 2005) and one of our previous studies (Zheng et al., 2018).

The *Home Sapiens* PR, androgen receptor (AR), α estrogen receptor (αER), mineralocorticoid receptor (MR) and glucocorticoid receptor (GR) sequences were downloaded from NCBI protein sequence database, and the alignment of these sequences were performed using T-Coffee alignment server (http://tcoffee.crg.cat/apps/tcoffee/index.html; Notredame et al., 2000), and the alignment figure was generated using BoxShade server (https://embnet.vital-it.ch/software/BOX_form.html).

The first hydration shell according to Sinha et al. (2008) is around 5 Å, therefore we used this distance as cutoff for calculating the coordination number between the protein (with ligand where necessary) and solvent water molecules. The water residence time was calculated based on the distance between a molecule and a protein residue within 5 Å during a continuous time, using a home-made python package (https://github.com/zhenglz/dockingML). Bridging water molecule was defined if a water molecule oxygen atom is within 0.35 nm distance range of both W755 sidechain nitrogen atom and V912 backbone nitrogen atom. The residence time of the bridging water molecule is calculated if it is continuously remaining close to the two residues for a period.

The electrostatic potential energies are calculated based on the following formula:

$$\begin{array}{l} E=\frac{q_{1}q_{2}}{4\pi \varepsilon_{0}d^{2}}, \end{array}$$

Where ε = 80 in this equation. And the electrostatic potential surface of the LBD were generated using Chimera (Pettersen et al., 2004) using default parameters. Here we only calculated the electrostatic interactions between fully solvent exposed residues (R899, K731, K734, K740, E723, E907, and E911), therefore, the water effect should be taken into account and a large ε = 80 was used.

## Results

### P4 Binding Reduces Agonistic PR LBD Conformation Flexibility

**Figure 1:**
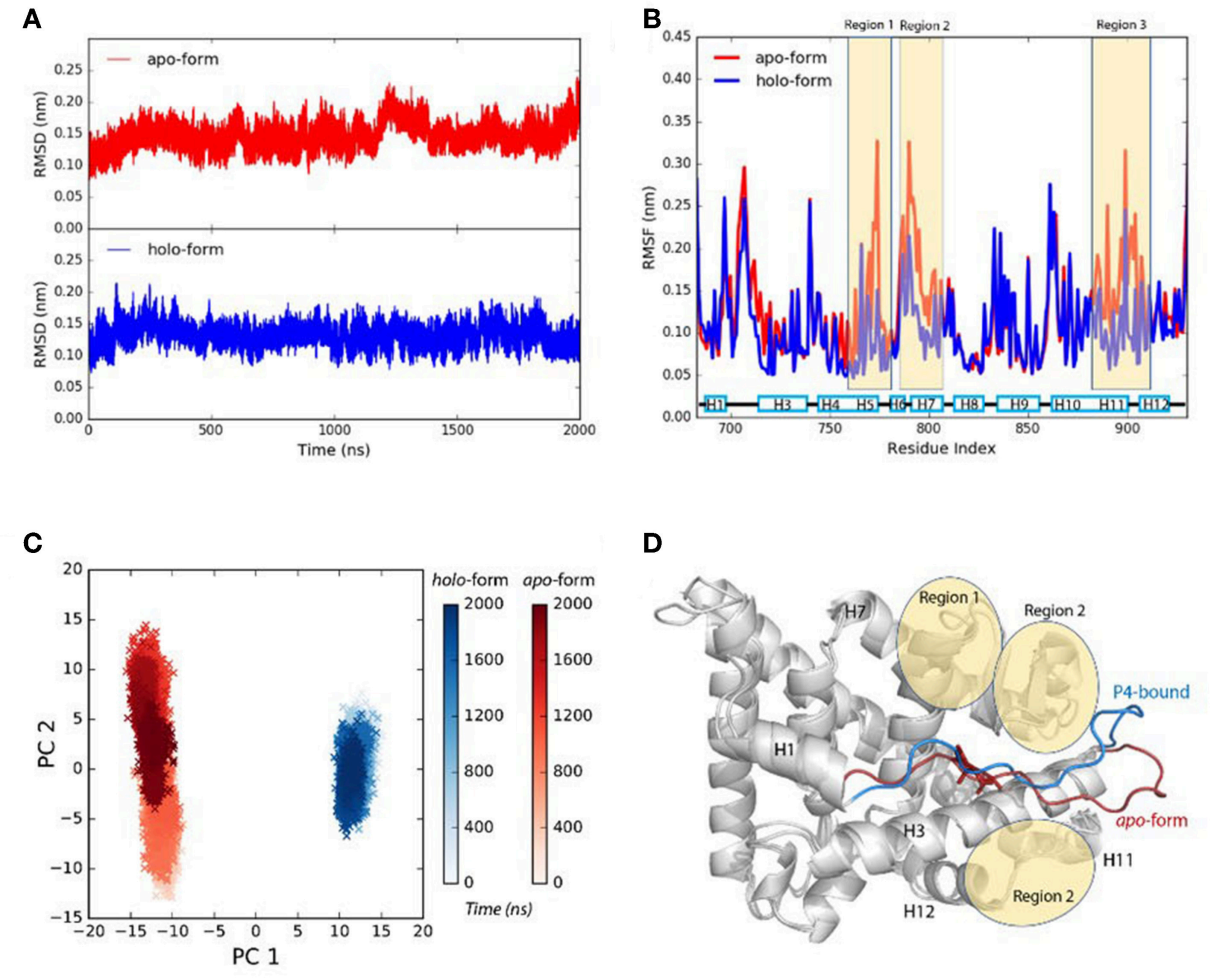
P4 binding stabilizes agonistic PR LBD conformation. **(A)** The αC atoms RMSDs of *apo*-form and P4-bound LBD. **(B)** The residue RMSFs of *apo*-form and P4-bound LBD. **(C)** PCA projections of contact matrix of αC atoms of *apo*-form and P4-bound LBD. **(D)** Structure alignment of the representative structures from *apo*-form and P4-bound LBD simulations. The *apo*-form and P4-bound LBD simulations are systems S1 and S2, respectively. The helices are labeled as “H,” for example, H12 in **(D)** (and all other figures where necessary) represents helix 12.

Agonist P4 binding would stabilize the residues around the binding pocket. (simulation S1, see experimental procedures) (Figure 1A). Three regions located around helix 5–7, helix 11 and 12, and the loops around these helices, are more flexible in P4-bound LBD (Figures 1B,C). And they have higher configurational entropy in *apo*-form LBD (1529.86 Jmol^−1^K^−1^) than P4-bound LBD (1230.43 Jmol^−1^K^−1^). Larger configurational entropy generally indicates a more flexible instinct. The evidence thus indicates that these residues could be stabilized with P4 binding.

Comparing the PCA projections of apo-form (simulation S1) and P4-bound (simulation S2) LBD simulations also indicate overall flexibility of the later system is more significant, as the projections on the PC2 are much broader for *apo*-form LBD than those for P4-bound *holo*-form LBD. The major configuration differences between unliganded and liganded LBD come from H1–H3 loop (the loop between helix 1 and helix 3) (Figure 1D). In P4-bound LBD, this loop shifts toward helix 3 and C-terminal of helix 11, and it binds tightly with region 1 and region 2 in P4-bound LBD majorly through electrostatic interactions and hydrogen bonds (Supplementary Figure 1).

In conclusion, the loss of P4 binding increases flexibility of residues around the LBP. H1–H3 loop shows different conformations in *apo*-form state and P4-bound state, though the biological significance of the dynamics of this loop remains unknown.

### P4 Binding Induced Helix 12 Conformational Adaptation Is a Multiple-Stage Process

It has been reported that induced fitting is quite a general mechanism in NR LBDs-ligand recognition (Bain et al., 2007), however, it is still unclear how agonists induce refolding of PR LBD. Our simulations indicate that binding of P4 results in the “open-to-closed” orientation rearrangement of helix 12, as well as the H11–H12 loop.

**Figure 2:**
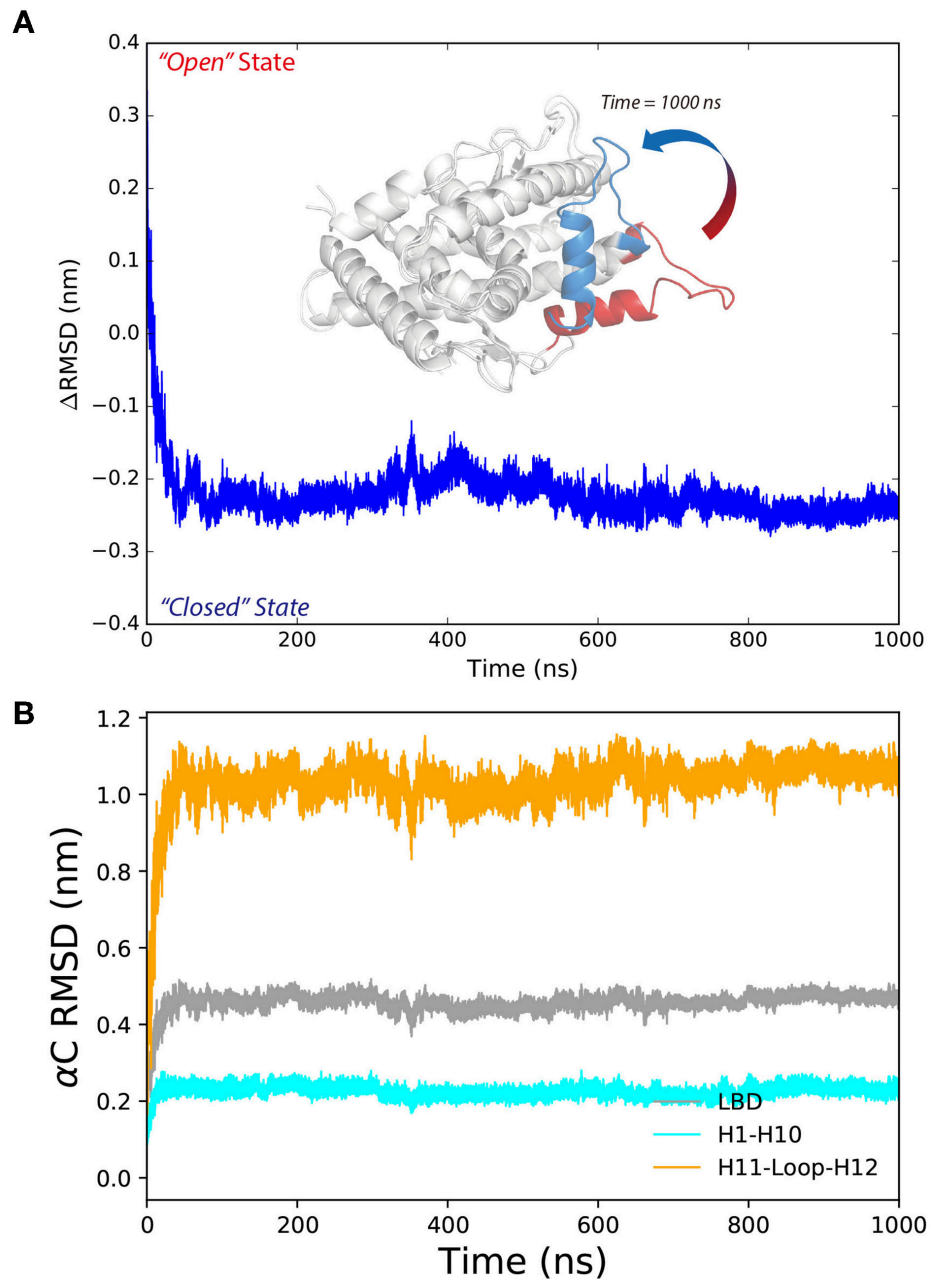
The conformational changes of antagonistic PR LBD with P4 binding. **(A)** The RMSD change along the simulation, where PR LBD adopts the “open” conformation at beginning and the “closed” conformation at the end. **(B)** The α-carbon RMSDs of different regions in PR LBD.

We use the *ΔRMSD* to assess how far the conformation is from the native “closed” state, and the smaller the value the more it is similar to the “closed” state. The *ΔRMSD* of the P4-bound antagonistic simulation (repeat #1 system S6) quickly dropped below −0.1 nm within the first 50 ns (Figure 2A). The decreasing of the *ΔRMSD* is majorly caused by the large conformational changes of helix 11, helix 12 and the H11–H12 loop (Figure 2B). Initially, helix 12 rapidly folds back and facilitates the LBD forming the “closed” state, which resembles the native agonistic “closed” state with a αC atoms RMSD < 0.2 nm (Figure 2A). The H11–H12 loop started to form contacts with P4 from around 300 ns. For example, L901 and M908 form non-polar contacts with the β11 position of P4 (Supplementary Figure 2). The contacts are also observed in crystal P4-bound PR LBD structure.

**Figure 3:**
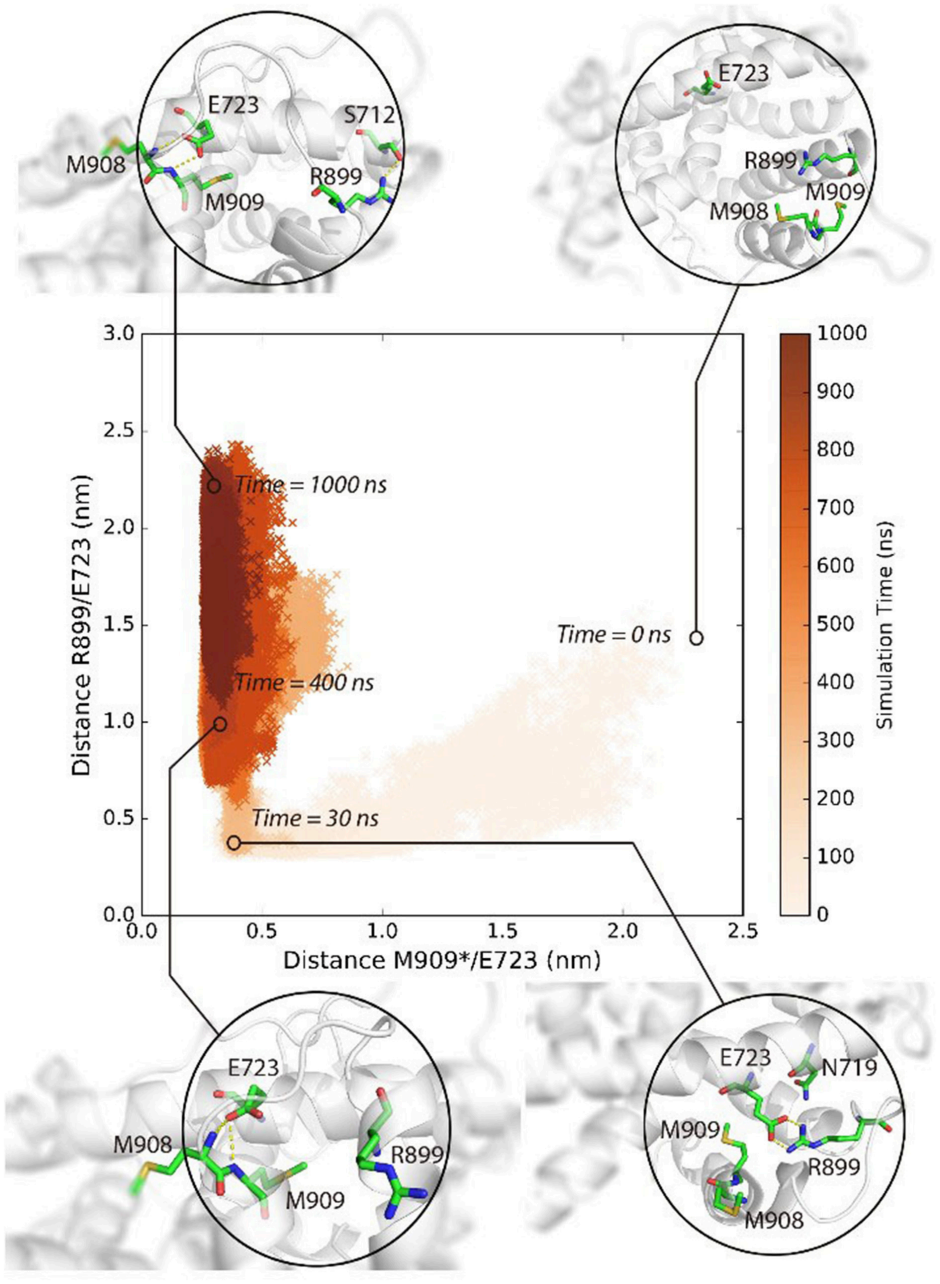
The conformational adaptation of antagonistic PR LBD with agonist P4 binding during the system S6 MD simulation. P4 was firstly docked into antagonistic PR LBD binding pocket. The MD simulations (simulation system S6) were then performed starting from the docked complex conformation. Several snapshots are presented here using cartoons.

M909 plays a vital role in stabilizing the agonistic state of PR LBD (Petit-Topin et al., 2009), and R899/E723 hydrogen bonds may affect the conformational dynamics of *apo*-form PR LBD (Zheng et al., 2016). These interactions would be good features to depict the conformational transition of P4-bound PR LBD. Initially, R899 is quite flexible and fully exposed to the solvent, and helix 12 remains the “open” orientation (Figure 3, time = 0 ns). The repositioning of helix 12 can be decomposed into several stages. For stage 1 (fast, from 0 ns to ~30 ns), R899 quickly approaches N719 and form transient contacts by hydrogen bonds, and then with E723 and forms hydrogen bonds by the sidechain oxygen atoms of E723. And V912, I913, W755, and P4 gradually form a hydrophobic core, thus restricting the flexibility of helix 12. This stage is characterized by the salt-bridges formation and the emergence of a hydrophobic core in the LBP.

As for stage 2 (from 30~50 ns), after the formation of electrostatic interactions and hydrogen bonds between E723 and R899, M909 sidechain starts inserting into the hydrophobic core and forms a hydrogen bond with the side chain of E723, resulting in dropping of distances between M909 and E723 within the first ~30 ns and remaining under 0.5 nm throughout the whole simulation, patching N-terminal end helix 12 tightly against helix 3 (Figure 3, time = 30 ns).

For stage 3, (after ~50 ns), R899 moves far apart from E723 and thus frees the restrictions around helix 11 C-terminal end (Figure 3, time = 400 ns). The re-orientation of R899 dismisses the space clash around helix 12 N-terminal end and enables the conformational adjustment of H11–H12 loop in later simulation, and helix 11 would be relatively more stable with the hydrogen bonds forming between R899 and S712 (Figure 3, time = 1,000 ns).

In summary, MD simulations reveal the multi-stage transition of PR LBD from “open” state to “closed” state: (1) fast hydrophobic core forming; (2) fast patching of helix 12 onto helix 3; and (3) slow adjustment of H11–H12 loop, highly coupling with key hydrogen bonds and distances of M909/E723 and R899/E723.

### PR LBD Forms the “Closed” Conformations Upon SPRM Binding

PR LBD may not adopt the antagonistic “open” state upon antagonists or SPRMs (such as RU486, asoprisnil) binding (Raaijmakers et al., 2009; Lusher et al., 2012), and helix 12 patches on the LBP and the LBD adopts the “closed” conformation as solved by soaking-based x-ray crystallography experiments, with helix 12 partially destabilized though (Lusher et al., 2012). The simulations (system S3, asoprisnil-bound PR LBD) recovered the SPRM-bound “closed” state PR LBD and found that helix 12 is less stable than when comparing to that in P4-bound LBD.

Initially, asoprisnil-bound LBD adopts the “open” conformation, and *ΔRMSD* is quite large. After 60 ns, the *ΔRMSD* decreases below −0.1 nm, and helix 12 blocks the pocket from the solvent access. Therefore, starting from the “open” conformation, asoprisnil-bound LBD quickly shifts toward the “closed” state, though the H11–H12 loop region is significantly disordered. Three independent repeat simulations both support the findings (Supplementary Figure 3).

The induced adaptation patterns were found to be similar to P4 induced conformational changes. During the early quick folding process, R899 approached N719 within a very short time scale (2 ns), their distance quickly drops from 2.0 to 0.3 nm. R899 forms salt bridges with E723 side chain and facilitates the formation of a hydrophobic core by L893, W755, V912, I913, and asoprisnil. Then the sidechain of M909, as well as that of M908, inserts into the hydrophobic core, leading to the patching of helix 12 onto helix 3 and the closing of the LBD.

The differences from P4-bound LBD case are that R899 interacts with the β11 oxygen atom of asoprisnil, and the interactions are rather stable and create steric hindrance for H11–H12 loop optimization, and the main chain of M909 does not form stable hydrogen bonds with E723 in the asoprisnil-bound case.

**Figure 4:**
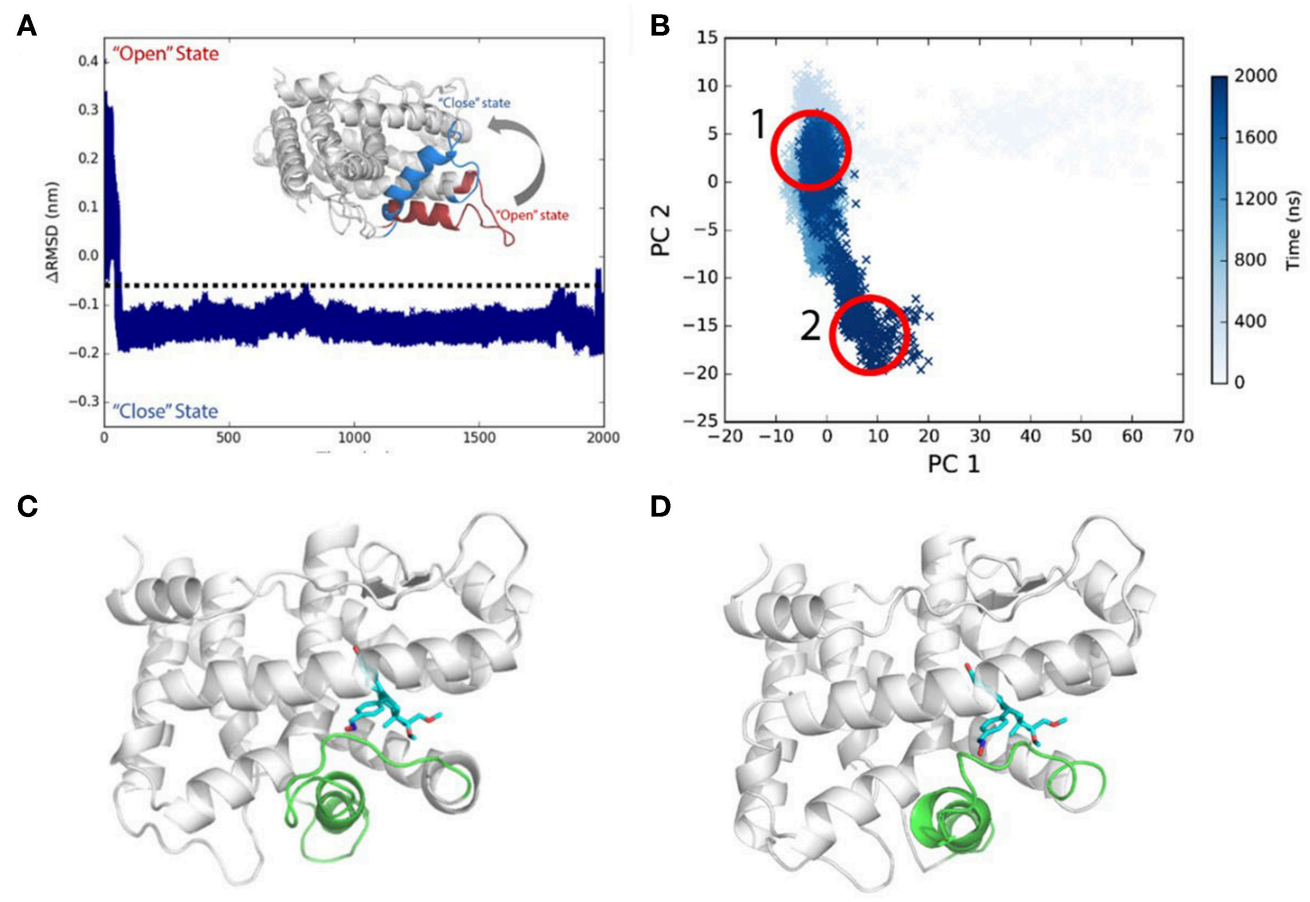
Conformational transitions of Asoprisnil bound PR LBD. **(A)** *ΔRMSD* of simulation system S3. **(B)** The PCA projections of the first two PCs using the coordinates. **(C)** Representative structure in PCA map region 1 in **(B)**. **(D)** Representative structure in PCA map region 2 in **(B)**.

Helix 12 remains relatively stable in the later simulation stage (after 100 ns). From PCA projections, it is clear that the system goes through a quick transition from “open” to “closed” state globally (Figures 4A,B). However, the diffusive spreading of the conformations along PC2, indicates that PR LBD does not have one to two major stable states, but rather it experiences structure adaptation. The representative structures have been extracted from regions 1 and 2 (in Figure 4B) showing a disordered H11–H12 loop (Figures 4C,D). We also performed metadynamics simulations to explore the free energy surface along the “open-to-closed” transition. The free energy difference between the “open” state and the “closed” state is around 25 kJ/mol (Supplementary Figure 4).

Overall, a SPRM binding would also induce conformational changes in helix 12, helix 11 and H11–H12 loop, though the H11–H12 loop remains disordered during the simulations (system S3). This disordering effect is rarely sampled in P4-bound LBD simulation (system S6). The conclusion is well consistent with that fact that in native antagonist-bound and SPRM-bound LBD structures [PDB ID: 1E3K (Matias et al., 2000), 1ZUC (Zhang et al., 2005), 1SQN (Madauss et al., 2004) and so on], helix 12 and H11–H12 loop are less ordered.

### Co-peptides Bound PR LBD Remains Unstable

Co-peptides binding would not stabilize the unliganded “open” state LBD (simulation system S5). The αC RMSD of PR LBD structure with reference to the native “open” state rises quickly (Supplementary Figure 5A). And the conformation of PR LBD also diverges from the agonistic “closed” state, where W755 and K919 sidechains remain close to each other.

**Figure 5:**
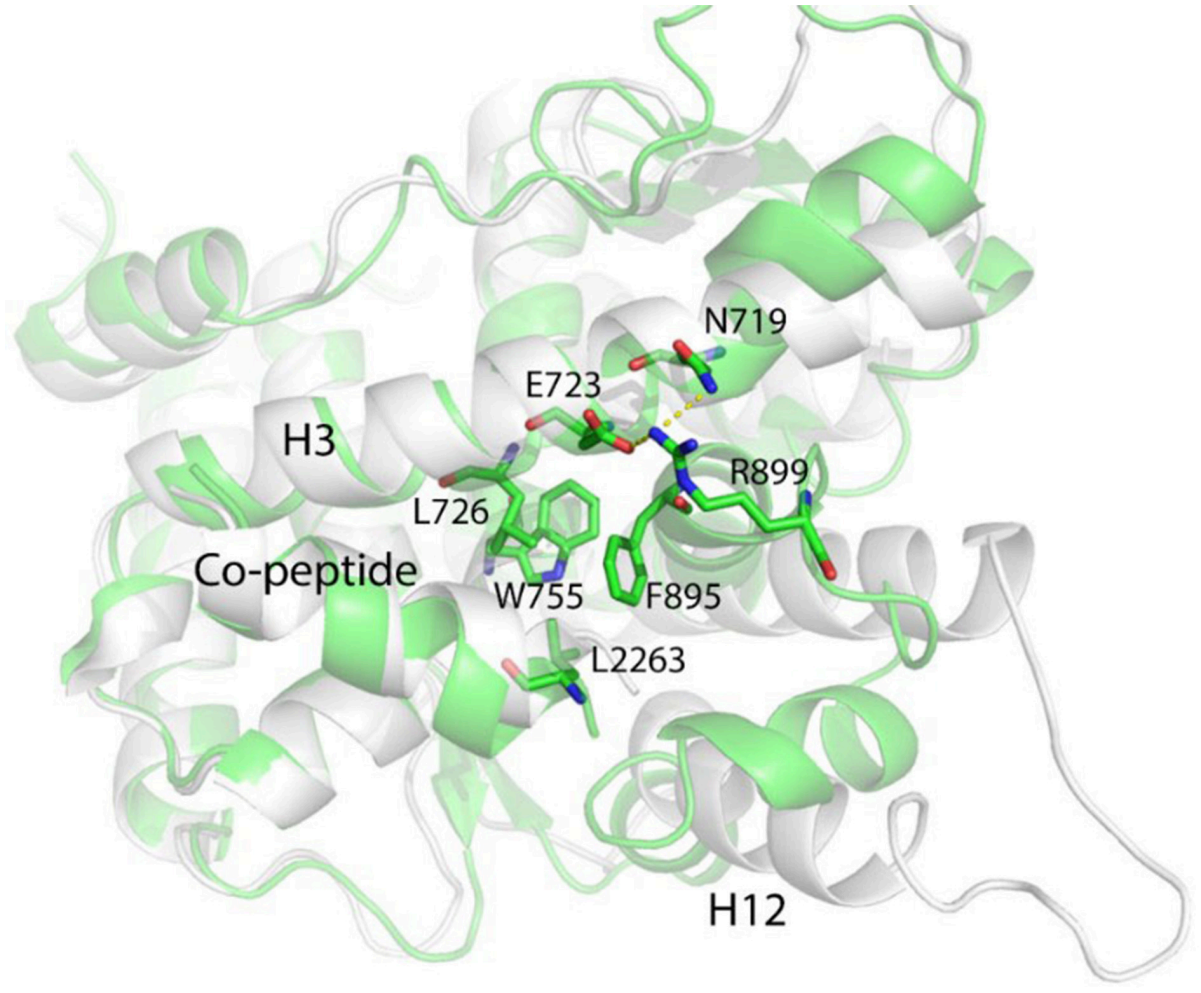
The representative conformation (green) of the largest population cluster superimposed with antagonistic PR LBD conformation (white).

W755 remains quite close to V912 for rather long simulation time, and E723 does not form any hydrogen bonds with M909 or M908. These evidences indicate that helix 12 C-terminal half part is restricted by the W755/V912 interactions and it takes the similar position as that in the native “closed” state, whereas N-terminal half remains flexible and exposed to the solvent. However, at late stage of the simulation (after 1500 ns), L2263 and I2267 in co-peptide gradually form hydrophobic interactions with W755 and push V912 in helix 12 away from W755 (Figure 5).

Helix 11 prohibits helix 12 patching and “closed” state formation by shifting toward the LBP and making contacts with the middle part of helix 3 through R899/E723 salt bridges (Figure 5). Helix 12 is almost identical to that in the “closed” state, characterized by a solvent exported K919, while in native “open” state LBD, K919 side chain is buried deeply in the LBP and forms a hydrogen bond with W755 side chain.

Overall, for unliganded LBD, if the co-peptide exists, the N-terminal part of helix 12 adopts the “open” state and is still quite flexible and the LBP is totally exposed to solvent. In the presence of both asoprisnil and the co-peptide binding, the antagonistic “open” conformation of the LBD is well maintained (Supplementary Figure 5B).

## Discussion

### Hydrophobic Effect Facilitates Helix 12 Re-packing

The hydrophobic effect is recognized as the dominant driving force for protein folding (Spolar et al., 1989; Dill, 1990; Callaway, 1994; Pace et al., 1996). Non-polar residues tend to be buried inside the protein core, inaccessible to solvent water molecules. With a ligand binding, the protein may become more stable or less stable and the ligand conformational entropy decrease would not be favorable for ligand-induced folding (Kauzmann, 1959; Levy and Onuchic, 2006). However, it has been proved that the translational entropy gains of water molecules escaping from protein surface would at least compensate that (Harano and Kinoshita, 2004; Kinoshita, 2009).

**Figure 6:**
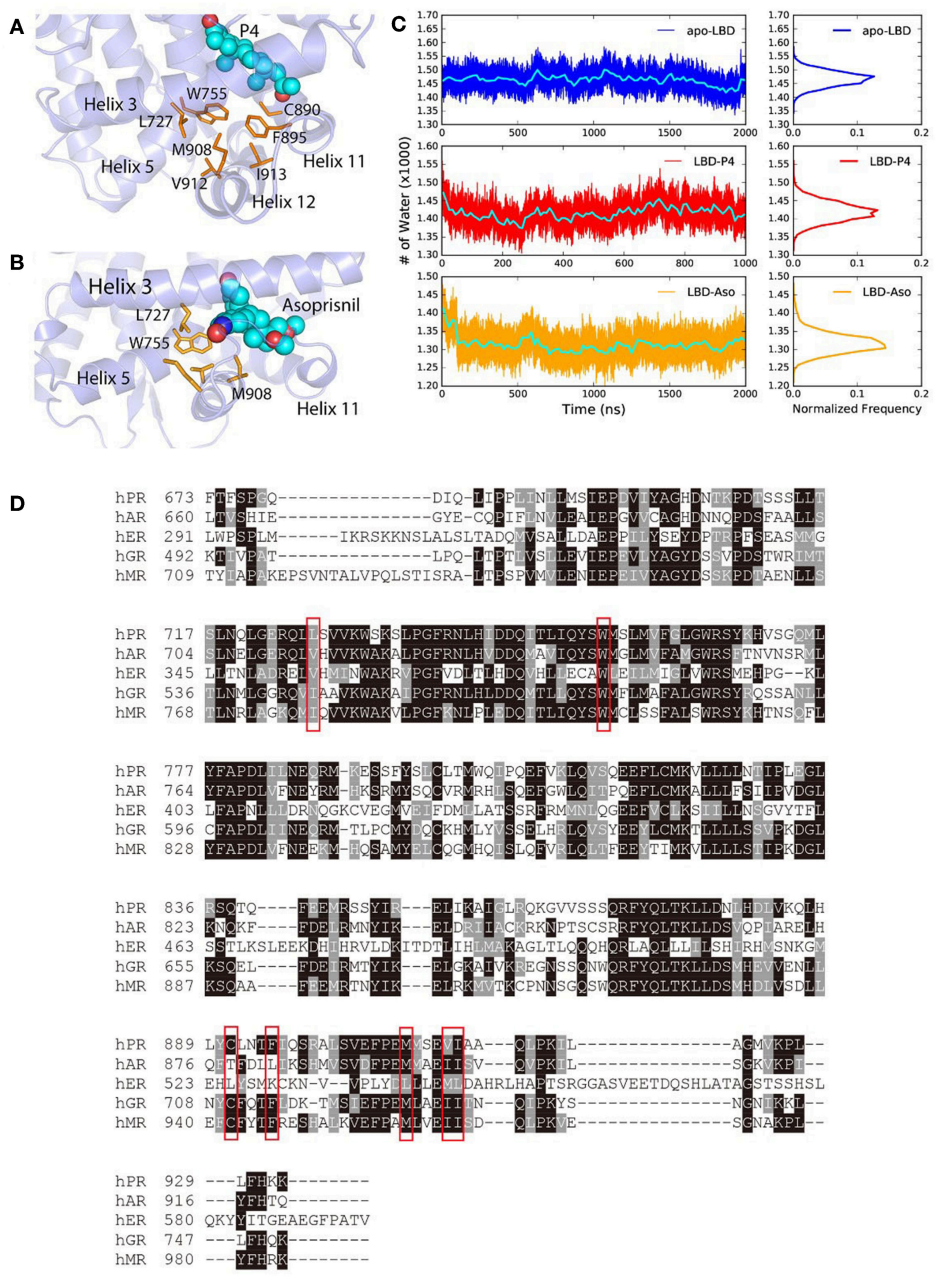
The hydrophobic cluster that may facilitate helix 12 patching. **(A)** *t* = 30 ns the hydrophobic cluster formed during simulation. **(B)** Asoprisnil-bound PR LBD hydrophobic core *t* = 60 ns. **(C)** Number of first hydration shell water molecules for *apo*-form “open” state LBD (system S4), P4-bound LBD (system S6), and asoprisnil-bound LBD (system S3). The blocked averages of the time series water numbers are shown in cyan lines. **(D)** Sequence alignment of PR, AR, αER, GR, and MR LBDs.

The steroids or steroid-like ligands are mostly hydrophobic (Williams and Sigler, 1998; Petit-Topin et al., 2009; Lusher et al., 2011) and the binding of these ligands would induce the forming of hydrophobic core and dehydration of the LBP, by re-orientating non-polar residues surrounding the pocket, therefore also facilitate the conformational changes of helix 11, helix 12, and H11–H12 loop in PR LBD. Such ligand-induced conformational changes were also observed in other NR LBDs (Mangelsdorf et al., 1995; Levenson et al., 2001; Huang et al., 2010). In the early stage of P4-bound induced conformational adaptation of PR LBD, the non-polar residues from helix 12 (M908, V912, and I913), helix 11 (C890 and F895), helix 5 (W755), as well as helix 3 (L727), together with the hydrophobic ligand P4, forms a hydrophobic cluster (Figure 6A), marking as the early event of the helix 12 re-orientation. Similarly, in asoprisnil-bound induced re-positioning of helix 12, these residues, except C890 and F895, also come close to each other and exclude the solvent molecules near the LBP (Figure 6B). Sequence alignment of several other NR LBDs indicates that these non-polar residues are quite conserved (Figure 6D). Especially, W755 is highly conserved in all five NR LBDs, suggesting the vital importance of this residue, while the other non-polar residues in PR LBD have their equivalent non-polar counterparts in αER, AR, MR, and GR LBDs.

We also observed the rapid loss of water molecules in the first hydration shell upon ligand-binding induced conformational changes. More specifically, coupling with the hydrophobic cluster formation, the number of 1^st^ layer hydration water molecules outside PR LBD surface decreases in the early stage of the “open-to-closed” transition (Figure 6C, Supplementary Figure 6). During the helix 12 reorientation for liganded LBD, more hydration water molecules are released from the protein surface.

To summarize, the ligand-induced formation of hydrophobic cluster by several conserved non-polar residues around the LBP and helix 12, is a key driving force for the conformational adaptation. Meanwhile, the loss of stabilized water molecules within the first hydration shell would also contribute to large favorable free energies to the helix 12 “open-to-closed” transition.

### Electrostatic Interactions Contribute to Helix 12 Patching

The short-range electrostatic interactions are important in protein folding and stability, and statistical analysis indicate that around 80% of the ion-pairs or polar-interactions of proteins are exposed to the solvent (Spolar et al., 1989). In PR LBD, we also identified several key short-range electrostatic interaction pairs or hydrogen bonds pairs.

Our previous simulation study suggested that E723 is actively involved the conformational dynamics of PR LBD (Zheng et al., 2016). Here, in this study, the highly frequent electrostatic interactions and hydrogen bonds between E723 and R899 are recorded in the early “open” to “close” transitions of P4-bound LBD and asoprisnil-bound LBD. The decrease of E723/R899 distance from around 1.5 nm to 0.3 nm would contribute to a favorable ~ 2.5 kJ/mol electrostatic energy change. Another hydrogen bond between H888/Q916 (formed in 67.9 and 18.2% of the snapshots of simulation systems S6 and S3, respectively) also stabilizes helix 12 in the “open-to-closed” transition of PR LBD. While the *ΔRMSD* of the ligand-induced LBD conformation decreases, the distance between H888/Q916 fluctuates in quite a large range (0.1 to 1.3 nm), indicating that local interactions around helix 12 changes accordingly (Supplementary Figure 7).

Furthermore, cancer genome sequencing studies showed the vital role of E723 whose E723K mutants detected in breast cancer patient tissues (Network, 2012), indicating the possible importance of the positive charge of this residue, as well as the electrostatic interactions for a functional PR LBD. However, the details had never been explained in atomic level elsewhere. In αER, an equivalent residue D351 also stabilizes helix 12 in the “closed” state LBD, the D351Y mutation and other artificial mutations would abolish the agonistic effect of αER (Webb et al., 2000; Levenson et al., 2001; Herynk and Fuqua, 2004; Lusher et al., 2012). Overall, we believe that E723 is an important residue for functional PR LBD possibly through electrostatic interactions.

Bridging water molecules are commonly involved in protein folding process (Levy and Onuchic, 2006). These water molecules would accelerate protein folding by taking a structural role, hydrogen bonding between two hydrophobic residues through their hydrophilic backbone atoms. However, in later stage these water molecules would be expelled from the hydrophobic core. These collective emptying of water molecules from hydrophobic core are quite common and the process is called dewetting (drying) effect, where the water molecules escape and large vapor bubbles form (Huang et al., 2003; Levy and Onuchic, 2006).

**Figure 7:**
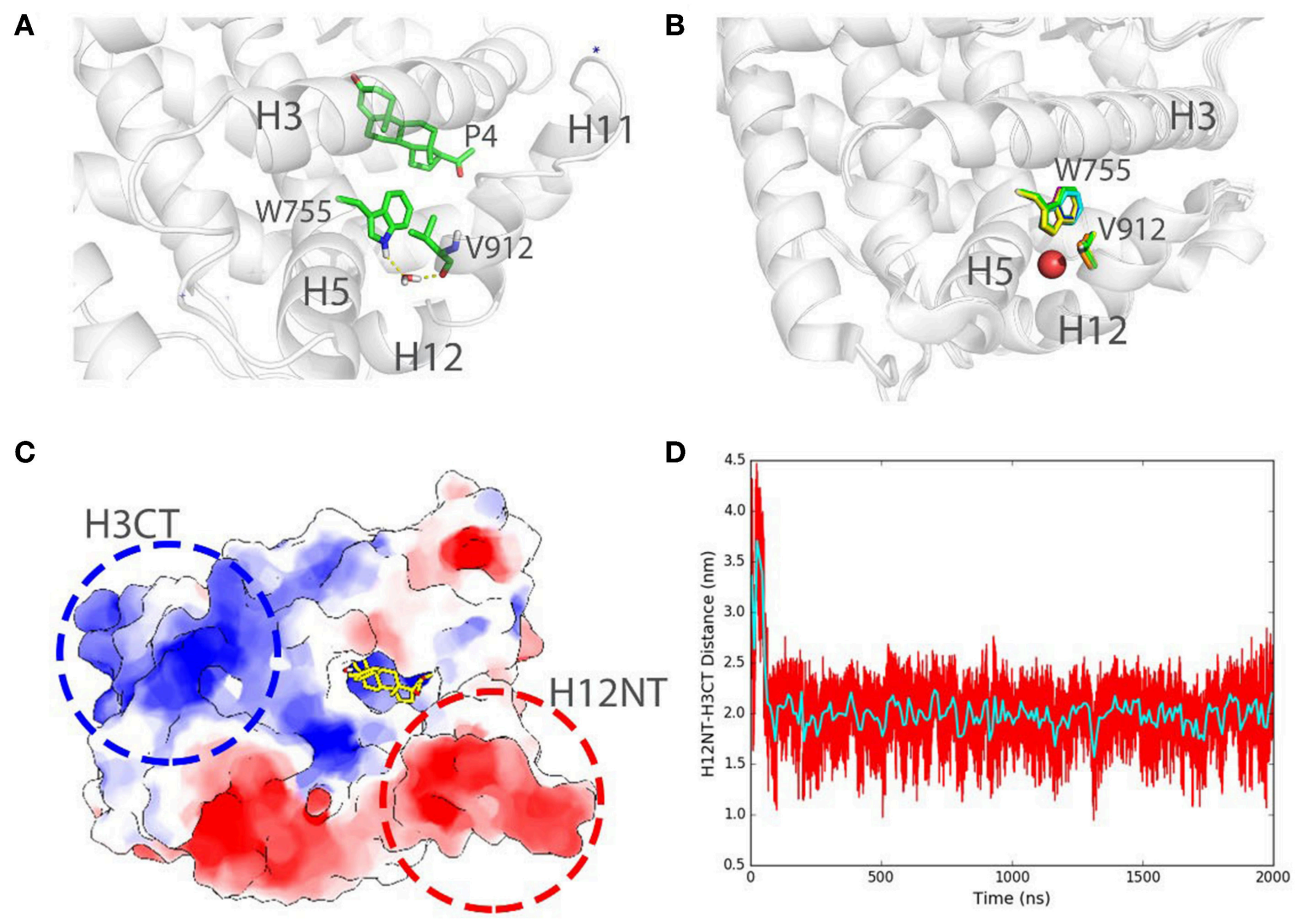
Structural water bridging helix 12 stabilization and long-range electrostatic interactions in PR LBD simulations. **(A)** Stable interaction formed between W755 and V912 bridging by a stable water molecule in P4-bound LBD simulation (system S6, *t* = 100 ns). **(B)** Structural alignment of several crystal structures of PR LBD with a conserved water molecule (red sphere) bridging W755/V912 interactions, the colors of the PDB structures are green for 1A28, blue for 1SQN, yellow for 2W8Y, magenta for 3D90, cyan for 3G80, orange for 3ZR7, and gray for 3ZRB. **(C)** A cartoon representation shows the two highly charged centers in PR LBD surface. **(D)** The distance between H12NT and H3CT during asoprisnil-bound LBD simulation, while the cyan line shows the time-blocked average of the distance. The blue asterisk indicates a close-contact sodium ion near the LBD.

We also record the existence of the structural water molecules in our simulations (Figure 7A) around W755 and V912. Interestingly, W755 captures V912 backbone oxygen atom in helix 12 indirectly, bridging by a well-conserved water molecule (Figure 7B), as also observed in several crystal structures of PR LBD [PDB ID: 1A28 (Williams and Sigler, 1998), 1SQN (Madauss et al., 2004), 2W8Y (Raaijmakers et al., 2009), 3D90 (Petit-Topin et al., 2009), 3G8O (Thompson et al., 2009), 3ZR7 (Lusher et al., 2011), and 3ZRB (Lusher et al., 2011)]. The residence time of this interior water molecule during the simulations is larger than 1 ns, which is correlated well with the experimental residence time of buried water molecules (Sinha et al., 2008). Since we removed all crystal water molecules at the beginning of the simulations, the resampling of the important structural water in the conserved crystal water position indicates that our simulations could capture key features of solvation dynamics around PR LBD, and that conserved structure water could facilitate the conformational stability of liganded PR LBD.

Furthermore, the long-range electrostatic interactions may also contribute to the helix 12 patching process. The N-terminal end of helix 12 (H12NT) is negatively charged (the negative charge center), mainly due to the existence of several polar residues E904, E907, and E911 (Figure 7C). The C-terminal end of helix 3 (H3CT), however, is highly positively charged (positive charge center) due to two polar residues, K733 and R740 (Figure 7C). The mutation of the positively charged residue R740, such as R740Q, would lead to breast cancer (Network, 2012). The energetic favorable long-range electrostatic attractions between the positive charge center and the negative charge center thus would further contribute to the “open” to “closed” transition. The distance between the positive charge center and the negative charge center shrinks from about 3.6 to 2.0 nm, and it would contribute to a favorable ~ 2.5 kJ/mol electrostatic potential energy change (Figure 7D).

These electrostatic interactions, hydrogen bonds and indirect water bridging, are crucial for PR LBD ligand-bound conformation reconfiguration and structural ability.

### Bulk 11β Group Blocks Stable Helix 12 Hooking and H11-H12 Loop Reordering

There has been discussed for long that M909 sidechain would sterically clash with the bulk 11β group on the steroid scaffold of PR antagonists (mifepristone) and SPRM asoprisnil (Fuhrmann et al., 2000; Leonhardt and Edwards, 2002; Raaijmakers et al., 2009; Lusher et al., 2011), which explains the partial agonism, or the loss of agonist response, of the molecules (Madauss et al., 2007; Petit-Topin et al., 2009; Huang et al., 2010; Lusher et al., 2011).

From the asoprisnil-bound simulations, it is clear that the bulk 11β group works as a double edge sword for PR LBD agonistic conformation formation and stabilization. In one way, it would facilitate the positioning of helix 12 to the agonistic state, by stabilizing the electrostatic interaction network formed by R899, E723, N719, and SPRM asoprisnil, and further restricting the motions of helix 11 C-terminal region. On the other hand, however, it makes clashes with the antagonist ligand and inhibits the close contacts between M909 and E723, therefore prohibits the hydrogen bonds formation and helix 12 conformational adaptation. M909 plays a vital role for the agonistic conformation stability as revealed in several studies (Madauss et al., 2007; Petit-Topin et al., 2009), it forms a hydrogen bond with E723 contributing a favorable interaction to the “closed” agonistic state, it also shields the LBP from solvent access. Thus, the inability to form this critical hydrogen bond would result in an unstable helix 12, and subsequently contribute to the partial agonism of asoprisnil-bound PR LBD (Petit-Topin et al., 2009; Raaijmakers et al., 2009; Huang et al., 2010).

The bulk 11β group destabilizes the agonistic contacts between H11–H12 loop with helix 3 as well. Hydrogen bonds formed between S712/L901, T716/E904, and N719/E904 are well maintained in agonistic conformation simulations (systems S1 and S2), but not in asoprisnil-bound LBD simulations (system S3). The observations are consistent with the evidence that larger 11β group would lead to destabilized helix 12 and H11–H12 loop (Raaijmakers et al., 2009; Lusher et al., 2012).

We believe that the bulk group in 11β position of steroid ligands hinders the occupation of the agonistic “closed” position for helix 12 and H11–H12 loop. Though SPRM asoprisnil also induces the conformational adaptation of helix 12, the space clashes contribute to the instability of this helix, as well as helix 11 and H11–H12 loop, which correlates with the finding of partial agonism of the SPRM ligands.

### Conformational Adaptions of PR LBD Upon a Ligand Binding

It has been shown that *apo*-form PR LBD is flexible and there exists multiple intermediates, among which the agonistic “closed” conformation is a rather stable state. Ligands (and/or peptides) binding, however, induces conformational changes or stabilizes one of the intermediates. From our simulations, we recorded the evidences of ligand and co-peptides binding-induced conformational adaptations.

**Figure 8:**
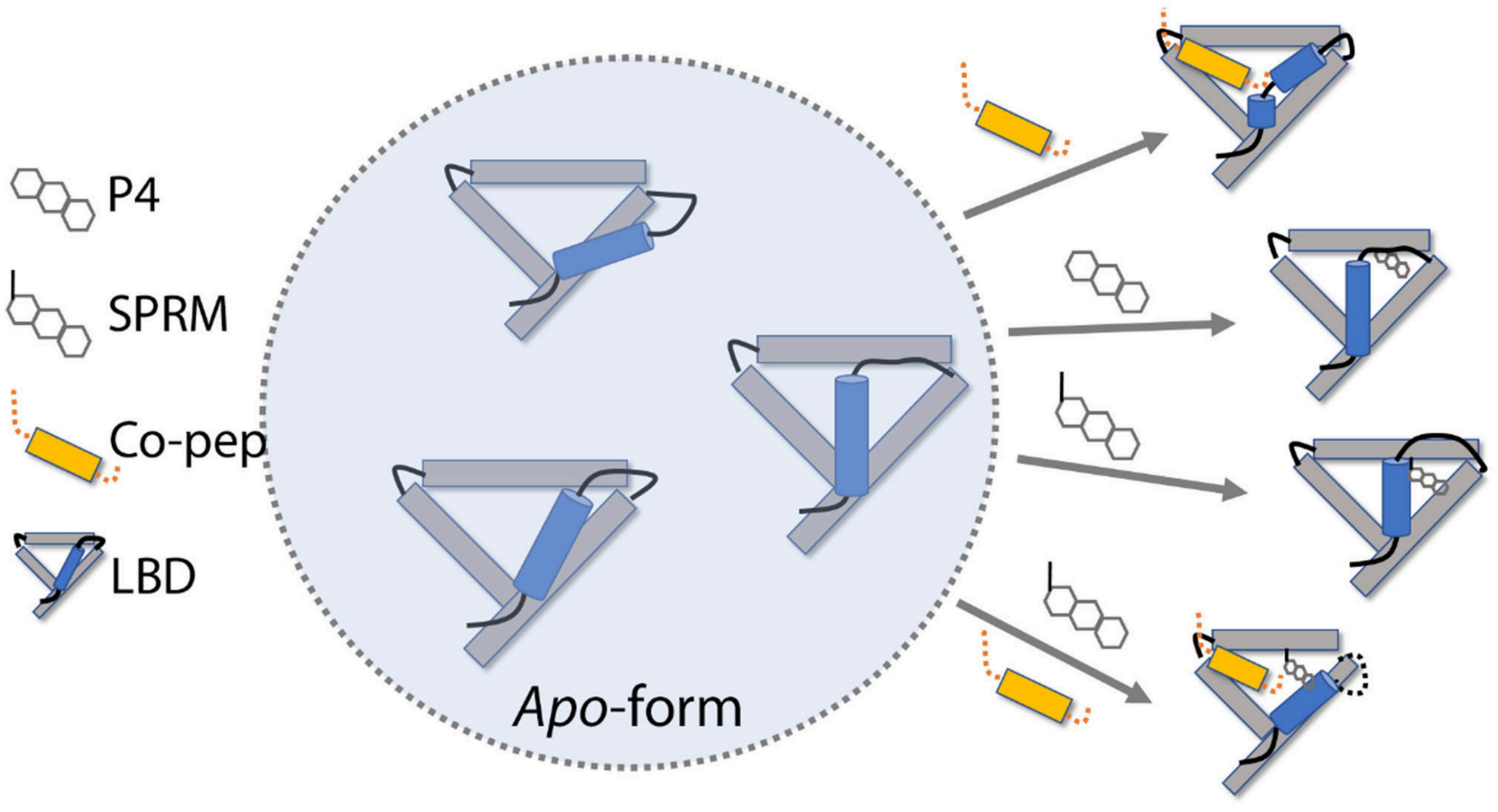
PR LBD conformational adaptations. SMRT co-repressor peptides are shown in orange. Only 4 helices (helices 3, 5, 11, and 12), as well as some loops, in the LBD are presented, whereas helix 12 is in ice-blue color. Key loops and tails in the LBD are shown as black lines.

Based on the simulation results, we propose a possible roadmap of PR LBD conformational adaptation induced by ligands or co-peptides (Figure 8). Firstly, both P4 and SPRM asoprisnil would induce the formation of agonistic-like “closed” state PR LBD, in which helix 12 covers the hydrophobic LBP. However, in SPRM-bound LBD, the “closed” state complex is not stable, helix 12 N-terminal end is not hooked by E723, and H11–H12 loop is not re-orientated to tightly patched against helix 3 in a short simulation period. These conformational adaptations thus explain the partial agonism of the SPRM (Leonhardt and Edwards, 2002; Chwalisz et al., 2005b). Secondly, with co-repressor peptides binding in the cleft formed by helix 3 and helix 5, helix 12 bends outwards, thus the PR LBD samples the semi-open antagonistic conformations, which would be favorable for both agonists or antagonists binding, but resists against co-activator binding (Germain et al., 2002). Thirdly, with both co-repressor peptides and the SPRM ligand binding, the antagonistic PR LBD conformations are well maintained. This is not surprising, since the SMRT co-repressor peptides could form ternary complex with RAR in complex with RAR-response element (Chen and Evans, 1995; Glass and Rosenfeld, 2000). The agonist binding induces releasing of SPRM co-repressor peptides, from RAR and thyroid-hormone receptor (TR; Chen and Evans, 1995), or possibly from PR, which may results from the spatial clashes between helix 12 and the SMRT co-repressor peptides (Madauss et al., 2007). And it is highly possible that there is a binding competition between co-repressor peptides and co-activator peptides (Glass and Rosenfeld, 2000; Germain et al., 2002; Madauss et al., 2007), and only with both SPRM and co-repressor peptides binding, PR LBD could adopt the antagonistic conformations. Meanwhile, an antagonist or SPRM ligand binding with LBD would facilitate the co-repressor peptides association and suppress the co-activator binding (Germain et al., 2002; Madauss et al., 2007).

However, there are still missing parts in this roadmap, such as how would the *apo*-form LBD react to the pure antagonistic binding? We do observe the destabilization of agonistic “closed” LBD with an antagonist (RU486) binding. We did not observe the transition of *apo*-form “closed” LBD to “open” state. The time scale of such transition may be beyond our simulation timescales. Besides, at moment we don't know how antagonism of antagonists is related to conformational changes of PR LBD. Further study of the conformational transition kinetics would be required to enhance our understanding of this PR LBD conformational adaptation roadmap.

## Data Availability

The datasets generated for this study can be found in Figshare, https://figshare.com/articles/Small_molecular_GAFF_force_field_files/7982333

## Author Contributions

LZ and YM designed the experiments. LZ, KX, and YM performed simulations and analyzed the data. LZ wrote the paper with YM and KX.

### Conflict of Interest Statement

The authors declare that the research was conducted in the absence of any commercial or financial relationships that could be construed as a potential conflict of interest.
