# Supplementary material for: Ligand Binding Induces Agonistic-Like Conformational Adaptations in Helix 12 of Progesterone Receptor Ligand Binding Domain
Source: Front Chem. 2019 May 7;7:315. doi: 10.3389/fchem.2019.00315 (PMC6514052; doi:10.3389/fchem.2019.00315)
Supplement: Supplementary file 1 [file Data_Sheet_1.PDF]

Supplementary Material

1 Supplementary Data

To explore the free energy surface of PR LBD with asoprisnil binding, we adopted well-tempered metadynamics simulations [1, 2] based on the path collective variables (CVs) [3]. Metadynamics simulations rely on adding adaptive Gaussian-like bias potential to specific pre-defined coordinates (CVs) to "push" the system from local minima to explore wider CV space. The re-weighting method thus is used to uncover the unbiased free energy surface based on the biased CVs or other unbiased CVs. The two-dimensional path CVs explore the conformational progress (S component) along the pre-defined transition pathway and the distance of a structure from the transition pathway (Z component). Here, we performed well-temper metadynamics [4] (with bias factor as 10.0) using Plumed 2.2.3 [5] patched Gromacs 5.1.2 [6]. The initial structure was taken from PDB ID 2OVH by removing the co-repressor peptides and adding the missing loop, using the same strategy as we adopted in a previous study [7]. A pre-defined 30 frames transition pathway was generated using iMOD server (http://imods.chaconlab.org/) [8], the detail procedure of the pathway definition could be found here [7]. For the pre-defined transition pathway, the first frame is the agonistic crystal structure PDB ID 1A28 chain A ("closed" state), while the last frame (30th frame) is the antagonistic/co-repressor peptides bound LBD ("open" state).

The sigma and gaussian width were 0.51 and 2,5 for path CV S, 0.0052 and 2.5 for path CV Z. The λ parameter was set as 920. The bias deposit pace was 500 steps. The same MD simulation protocols and parameters were used as described in the Materials and Methods part. The one-dimensional free energy surface curve along path CV S was calculated using plumed sum_hills module. Multiple free energy surfaces were generated from the beginning of the simulation to the end of simulation. To evaluate the convergence of the simulation, the 9 free energy surfaces were recovered from 0 ns to 430 ns, 440 ns, 450 ns, 460 ns, 470 ns, 480 ns, 490 ns, 500 ns and 510 ns respectively. (Supplementary Figure 4)

2 Supplementary Figures and Tables

2.1 Supplementary Figures

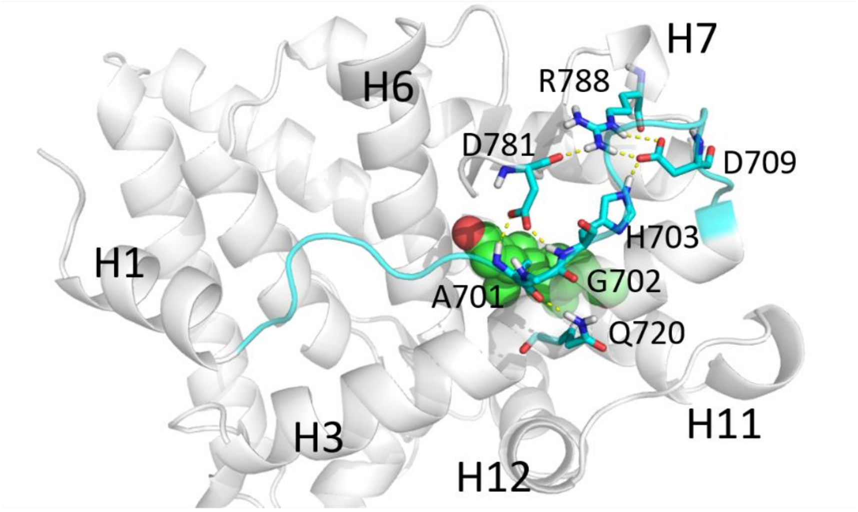

Supplementary Figure 1. The hydrogen bonding network between H1-H3 loop and flexible loops around H6 and H7. Hydrogen bonds are shown as yellow dashed lines. H1-H3 loop is cyan.

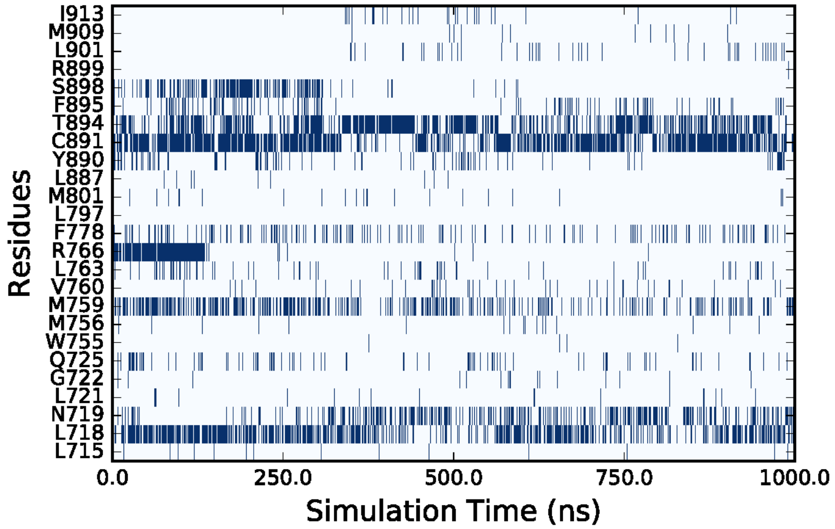

Supplementary Figure 2. The LDB-P4 residue interaction patterns along the simulation (system S6, in Table 1). The contacts are indicated by blue bars, while white bars indicate that no contacts exist.

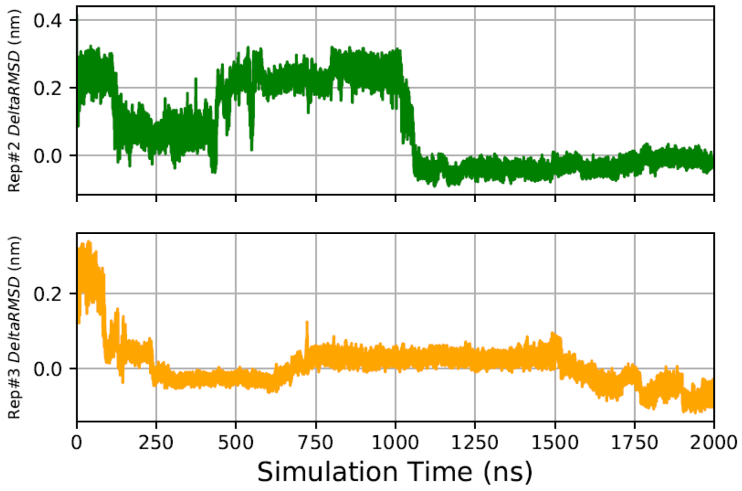

Supplementary Figure 3. The *ΔRMSD*s of the two simulations (repeat simulation 2 and 3) of system S3.

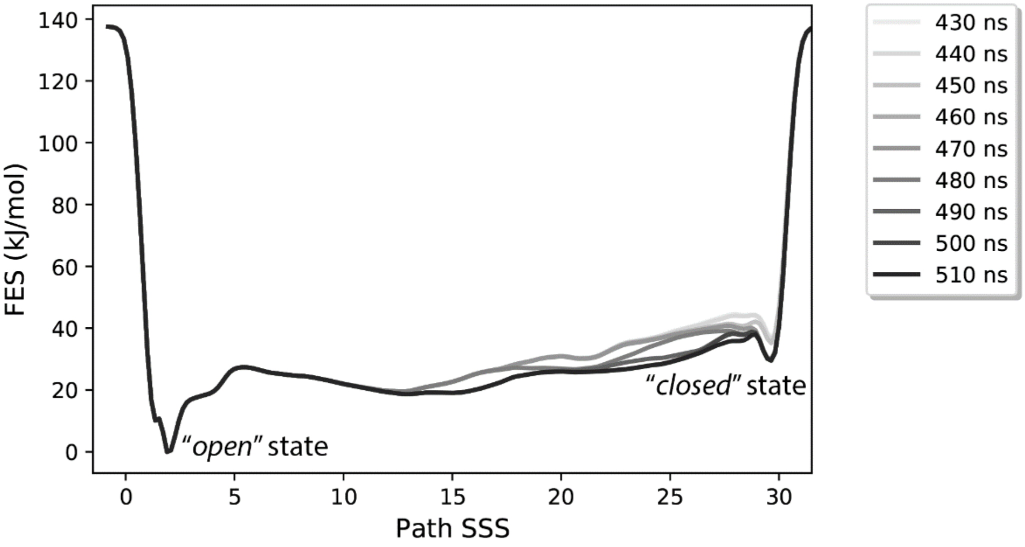

Supplementary Figure 4. The free energy surfaces (FES) along the one-dimensional path CV S recovered from well-tempered metadynamics simulations of the LBD-asoprisnil complex. The *x*-axis indicates the transition progress along path CV S from the "closed" state (path SSS = 0) to "open" state (path SSS = 30).

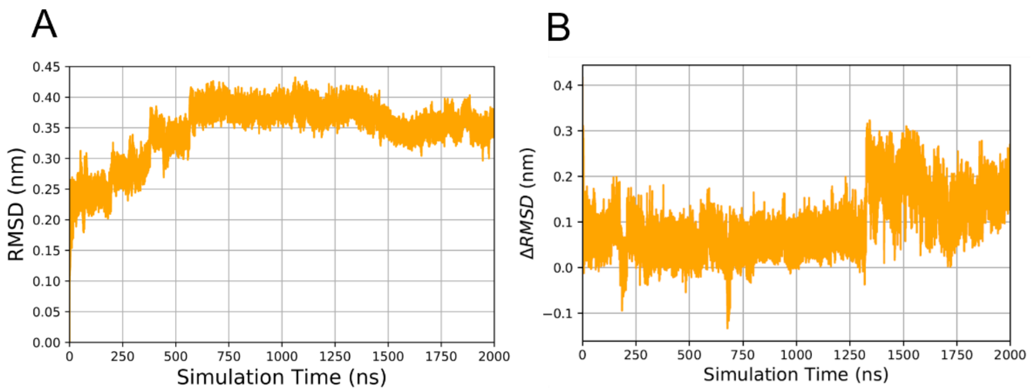

Supplementary Figure 5. A) the RMSD of the αC atoms of PR LBD in simulation S5. B) *ΔRMSD* of LBD/asoprisnil/co-peptides (simulation system S7) along the simulation.

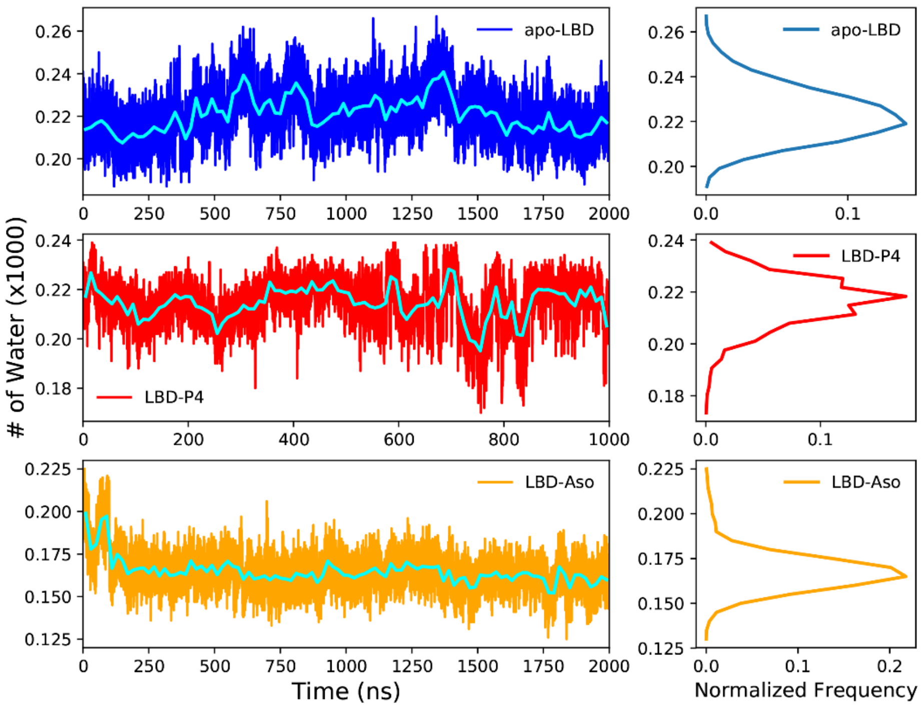

Supplementary Figure 6. The number of first hydration shell water molecules around helix 12 for *apo*-form “open” state LBD (system S4), P4-bound LBD (system S6), and asoprisnil-bound LBD (system S3). The blocked averages of the time series water numbers are shown in cyan lines.

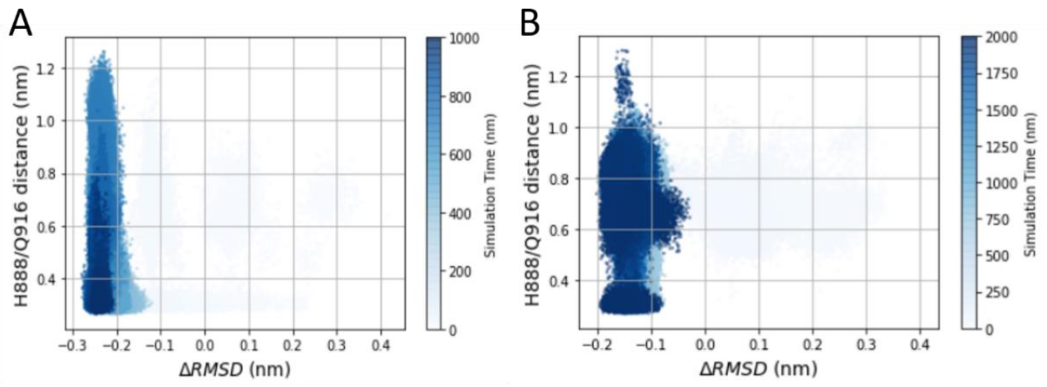

Supplementary Figure 7. The heatmaps of scatter plots of *ΔRMSD* and ND1@H888/ NE2@Q916 distance. A), simulation system S6. B), simulation system S3.

Reference

1. Barducci, A., M. Bonomi, and M. Parrinello, *Metadynamics.* Wiley Interdisciplinary Reviews: Computational Molecular Science, 2011. **1**(5): p. 826-843.
2. Tiwary, P. and M. Parrinello, *From metadynamics to dynamics.* Physical review letters, 2013. **111**(23): p. 230602.
3. Branduardi, D., F.L. Gervasio, and M. Parrinello, *From A to B in free energy space.* The Journal of chemical physics, 2007. **126**(5): p. 054103.
4. Barducci, A., G. Bussi, and M. Parrinello, *Well-tempered metadynamics: a smoothly converging and tunable free-energy method.* Physical review letters, 2008. **100**(2): p. 020603.
5. Tribello, G.A., et al., *PLUMED 2: New feathers for an old bird.* Computer Physics Communications, 2014. **185**(2): p. 604-613.
6. Abraham, M.J., et al., *GROMACS: High performance molecular simulations through multi-level parallelism from laptops to supercomputers.* SoftwareX, 2015. **1**: p. 19-25.
7. Zheng, L.Z., V.C. Lin, and Y.G. Mu, *Exploring Flexibility of Progesterone Receptor Ligand Binding Domain Using Molecular Dynamics.* Plos One, 2016. **11**(11).
8. López-Blanco, J.R., et al., *iMODS: internal coordinates normal mode analysis server.* Nucleic acids research, 2014. **42**(W1): p. W271-W276.
